# (Benzoato-κ*O*)(benzoic acid-κ*O*)(4,4′-dimethyl-2,2′-bipyridine-κ^2^ *N*,*N*′)hydroxidocopper(II) monohydrate

**DOI:** 10.1107/S1600536809043189

**Published:** 2009-11-07

**Authors:** Li Yao, Wen-Juan Li

**Affiliations:** a School of Computer and Information Engineering, Henan University, Kaifeng 475001, Henan, People’s Republic of China; b Department of Civil and Environmental Engineering, East China Institute of Technology, 56 Xuefu Road, Fuzhou 344000, Jiangxi, People’s Republic of China

## Abstract

In the structure of the title complex, [Cu(C_7_H_5_O_2_)(OH)(C_12_H_12_N_2_)(C_7_H_6_O_2_)]·H_2_O, the Cu^II^ ion is penta­coordinated in a tetra­gonal-pyramidal geometry with one O atom of a hydroxide group, one O atom of a benzoate anion and two N atoms of a 4,4′-dimethyl-2,2′-bipyridine ligand occupying the basal plane, and one O atom of a benzoic acid mol­ecule located at the apical site. The title complex was refined with a metal-coordinated OH group and a ‘free’ benzoic acid molecule, although it can be assumed that the proton is delocalized between the OH and the COOH group. The uncoordinated water mol­ecule is equally disordered over two positions. The structure displays O—H⋯O hydrogen bonding.

## Related literature

For selected 4,4′-dimethyl-2,2′-bipyridine copper complexes, see: Deschamps *et al.* (2002 ▶); Dong *et al.* (2006 ▶); Feng *et al.* (2007 ▶); Lin *et al.* (2008 ▶); Qian & Huang (2006 ▶); Willett *et al.* (2001 ▶).
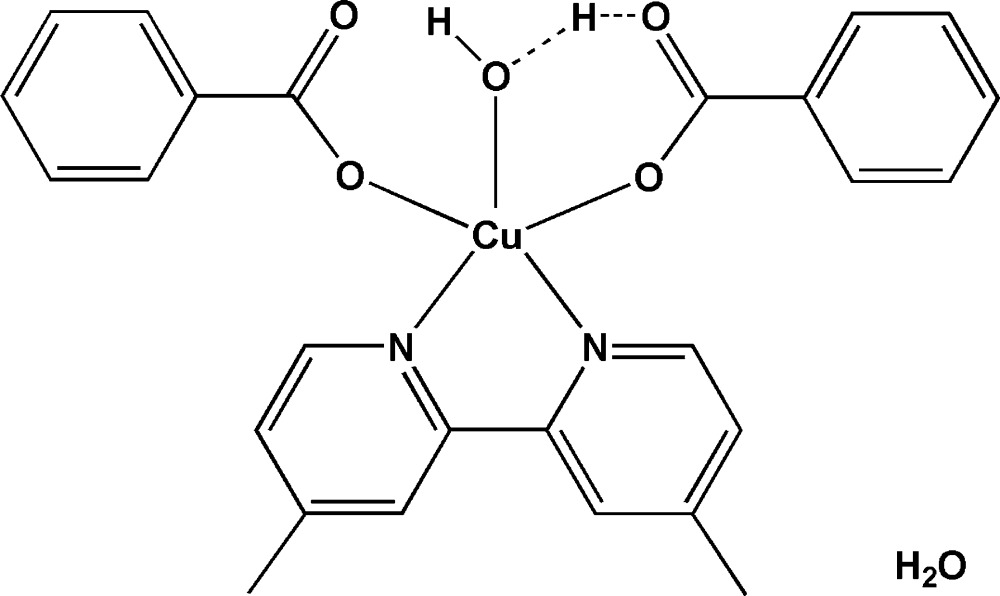


## Experimental

### 

#### Crystal data

[Cu(C_7_H_5_O_2_)(OH)(C_12_H_12_N_2_)(C_7_H_6_O_2_)]·H_2_O
*M* *_r_* = 526.03
Monoclinic, 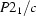

*a* = 11.3325 (15) Å
*b* = 17.155 (2) Å
*c* = 13.4007 (18) Å
β = 98.049 (3)°
*V* = 2579.5 (6) Å^3^
*Z* = 4
Mo *K*α radiation
μ = 0.89 mm^−1^
*T* = 296 K
0.28 × 0.26 × 0.25 mm

#### Data collection

Bruker SMART APEXII CCD area-detector diffractometer
Absorption correction: multi-scan (*SADABS*; Bruker, 2005 ▶) *T*_min_ = 0.789, *T*_max_ = 0.808
13741 measured reflections
4538 independent reflections
3075 reflections with *I* > 2σ(*I*)
*R*_int_ = 0.053

#### Refinement

*R*[*F* ^2^ > 2σ(*F* ^2^)] = 0.053
*wR*(*F* ^2^) = 0.146
*S* = 0.95
4538 reflections
329 parameters
H-atom parameters constrained
Δρ_max_ = 0.53 e Å^−3^
Δρ_min_ = −0.25 e Å^−3^

### 

Data collection: *APEX2* (Bruker, 2005 ▶); cell refinement: *SAINT* (Bruker, 2005 ▶); data reduction: *SAINT*; program(s) used to solve structure: *SHELXS97* (Sheldrick, 2008 ▶); program(s) used to refine structure: *SHELXL97* (Sheldrick, 2008 ▶); molecular graphics: *SHELXTL* (Sheldrick, 2008 ▶); software used to prepare material for publication: *SHELXTL*.

## Supplementary Material

Crystal structure: contains datablocks I, global. DOI: 10.1107/S1600536809043189/zq2009sup1.cif

Structure factors: contains datablocks I. DOI: 10.1107/S1600536809043189/zq2009Isup2.hkl

Additional supplementary materials:  crystallographic information; 3D view; checkCIF report

## Figures and Tables

**Table 1:** Hydrogen-bond geometry (Å, °)

*D*—H⋯*A*	*D*—H	H⋯*A*	*D*⋯*A*	*D*—H⋯*A*
O2*W*—H2*WB*⋯O2^i^	0.85	2.51	3.195 (8)	138
O2*W*—H2*WA*⋯O1*W*	0.85	2.24	2.821 (10)	125
O1*W*—H1*WB*⋯O4	0.85	2.26	2.932 (6)	136
O1*W*—H1*WA*⋯O2*W* ^ii^	0.85	2.28	2.937 (10)	134
O5—H5*A*⋯O4	0.82	1.93	2.642 (4)	145
O2—H2⋯O5	0.82	1.86	2.636 (5)	158

Symmetry codes: (i) 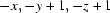; (ii) 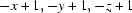.

**Fig. 1.**
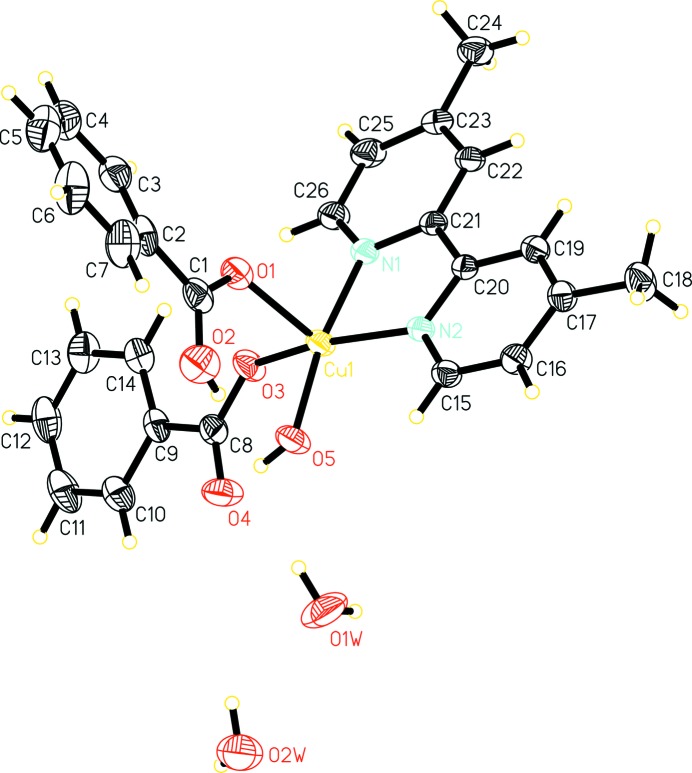
The molecular structure of the title compound with the atom-labelling scheme. Displacement ellipsoids are drawn at the 30% probability level. H atoms are presented as small spheres of arbitrary radius.

**Fig. 2.**
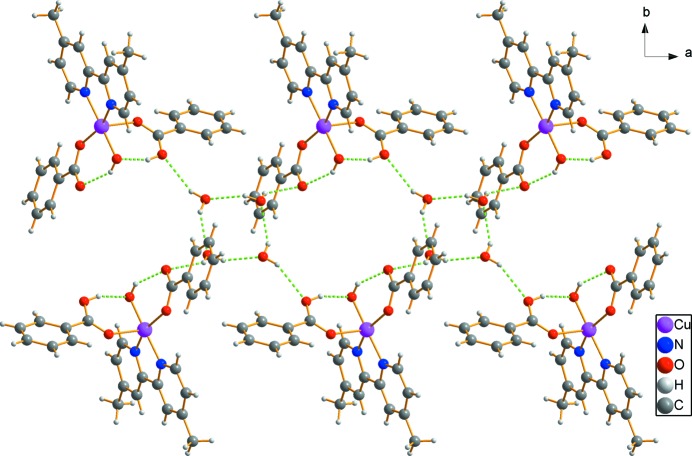
Crystal packing of the title compound. Hydrogen-bond interactions are drawn with dashed lines.

## supplementary crystallographic information

### Comment

As a contribution to structural characterization of 4,4'-dimethyl-2,2'-bipyridine copper complexes (Deschamps *et al.*, 2002; Dong *et al.*, 2006; Feng *et al.*, 2007; Lin *et al.* 2008; Qian & Huang, 2006; Willett *et al.*, 2001), we present here the crystal structure of the title complex, [Cu*LL*'*L*''(OH)].H_2_O (*L *= benzoate, *L*' = benzoic acid, *L*'' = 4,4'-dimethyl-2,2'-bipyridine).

In the structure of the title complex, the short O2···O5 separation of 2.636 (5) Å clearly indicates a typical hydrogen bond. The corresponding hydrogen atom was located in the Fourier difference maps near O2 (hydroxido benzoic acid type complex A) although general chemical considerations would rather expect it on O5 (water benzoate type complex B). One can assume that the proton is delocalised somewhere in-between as presented in Scheme 1 but based on the X-ray data only, the title complex was finally refined with a metal-coordinated OH group and a "free" benzoic acid molecule.

In the complex, the Cu^2+^ ion is pentacoordinated, with two N atoms of 4,4'-dimethyl-2,2'-bipyridine, one O atom of a hydroxide group and one O atom of a benzoate anion in the basal plane and one O atom of a benzoic acid molecule completing the square-pyramidal geometry from the apical site (Fig. 1). The atoms N1, N2, O1 and O3 are nearly coplanar, and the Cu atom is displaced by 0.2309 (5) Å from this plane towards the apical O atom. The water solvent molecule is disordered over two positions in a 1:1 ratio.

With O—H···O hydrogen bonds (Table 1), an one-dimensional chain is formed as shown in Fig. 2.

### Experimental

The title compound was synthesized hydrothermally in a Teflon-lined autoclave (25 ml) by heating a mixture of 4,4'-dimethyl-2,2'-bipyridine (0.2 mmol), benzoic acid (0.4 mmol) and CuSO_4_.5H_2_O (0.2 mmol) in water (10 ml) at 393 K for 3 d. Suitable crystals for an X-ray analysis were obtained.

### Refinement

All H atoms were included in calculated positions, with C—H bond lengths fixed at 0.96 Å (methyl CH_3_), 0.93 Å (aryl group) and O—H = 0.85 Å and were refined in the riding-model approximation. *U*_iso_(H) values were calculated at 1.5 *U*_eq_(C) for methyl H atoms and 1.2 *U*_eq_(C) for the other H atoms.

### Crystal data

**Table:**

[Cu(C_7_H_5_O_2_)(OH)(C_12_H_12_N_2_)(C_7_H_6_O_2_)]·H_2_O	*F*(000) = 1092
*M**_r_* = 526.03	*D*_x_ = 1.355 Mg m^−^^3^
Monoclinic, *P*2_1_/*c*	Mo *K*α radiation, λ = 0.71073 Å
Hall symbol: -P 2ybc	Cell parameters from 3905 reflections
*a* = 11.3325 (15) Å	θ = 2.8–24.2°
*b* = 17.155 (2) Å	µ = 0.89 mm^−^^1^
*c* = 13.4007 (18) Å	*T* = 296 K
β = 98.049 (3)°	Block, colourless
*V* = 2579.5 (6) Å^3^	0.28 × 0.26 × 0.25 mm
*Z* = 4	

### Data collection

**Table:**

Bruker SMART APEXII CCD area-detector diffractometer	4538 independent reflections
Radiation source: fine-focus sealed tube	3075 reflections with *I* > 2σ(*I*)
graphite	*R*_int_ = 0.053
φ and ω scans	θ_max_ = 25.0°, θ_min_ = 1.8°
Absorption correction: multi-scan (*SADABS*; Bruker, 2005)	*h* = −13→13
*T*_min_ = 0.789, *T*_max_ = 0.808	*k* = −20→11
13741 measured reflections	*l* = −14→15

### Refinement

**Table:**

Refinement on *F*^2^	Primary atom site location: structure-invariant direct methods
Least-squares matrix: full	Secondary atom site location: difference Fourier map
*R*[*F*^2^ > 2σ(*F*^2^)] = 0.053	Hydrogen site location: inferred from neighbouring sites
*wR*(*F*^2^) = 0.146	H-atom parameters constrained
*S* = 0.95	*w* = 1/[σ^2^(*F*_o_^2^) + (0.0932*P*)^2^] where *P* = (*F*_o_^2^ + 2*F*_c_^2^)/3
4538 reflections	(Δ/σ)_max_ < 0.001
329 parameters	Δρ_max_ = 0.53 e Å^−^^3^
0 restraints	Δρ_min_ = −0.25 e Å^−^^3^

### Special details

**Table:**

Geometry. All e.s.d.'s (except the e.s.d. in the dihedral angle between two l.s. planes) are estimated using the full covariance matrix. The cell e.s.d.'s are taken into account individually in the estimation of e.s.d.'s in distances, angles and torsion angles; correlations between e.s.d.'s in cell parameters are only used when they are defined by crystal symmetry. An approximate (isotropic) treatment of cell e.s.d.'s is used for estimating e.s.d.'s involving l.s. planes.
Refinement. Refinement of *F*^2^ against ALL reflections. The weighted *R*-factor *wR* and goodness of fit *S* are based on *F*^2^, conventional *R*-factors *R* are based on *F*, with *F* set to zero for negative *F*^2^. The threshold expression of *F*^2^ > σ(*F*^2^) is used only for calculating *R*-factors(gt) *etc*. and is not relevant to the choice of reflections for refinement. *R*-factors based on *F*^2^ are statistically about twice as large as those based on *F*, and *R*- factors based on ALL data will be even larger.

### Fractional atomic coordinates and isotropic or equivalent isotropic displacement parameters (Å^2^)

**Table:**

	*x*	*y*	*z*	*U*_iso_*/*U*_eq_	Occ. (<1)
Cu1	0.09687 (4)	0.20024 (2)	0.53039 (3)	0.05062 (19)	
N1	0.1716 (2)	0.09925 (16)	0.5797 (2)	0.0480 (7)	
N2	0.0537 (2)	0.13606 (16)	0.40610 (19)	0.0450 (7)	
O1	−0.0650 (2)	0.18703 (15)	0.6049 (2)	0.0656 (7)	
O2	−0.1757 (3)	0.2835 (2)	0.5320 (3)	0.0854 (9)	
H2	−0.1116	0.2989	0.5178	0.128*	
O3	0.1942 (2)	0.25605 (15)	0.63954 (18)	0.0631 (7)	
O4	0.1932 (3)	0.37656 (17)	0.5773 (2)	0.0797 (9)	
C1	−0.1583 (4)	0.2267 (3)	0.5918 (3)	0.0622 (10)	
C2	−0.2573 (3)	0.2055 (2)	0.6527 (3)	0.0654 (11)	
C3	−0.2304 (4)	0.1691 (3)	0.7438 (3)	0.0709 (11)	
H3	−0.1516	0.1562	0.7670	0.085*	
C4	−0.3181 (5)	0.1511 (3)	0.8019 (4)	0.0877 (14)	
H4	−0.2984	0.1260	0.8635	0.105*	
C5	−0.4335 (5)	0.1702 (4)	0.7687 (4)	0.0992 (17)	
H5	−0.4922	0.1588	0.8085	0.119*	
C6	−0.4635 (4)	0.2054 (4)	0.6789 (4)	0.1010 (19)	
H6	−0.5429	0.2172	0.6567	0.121*	
C7	−0.3759 (4)	0.2245 (3)	0.6186 (4)	0.0898 (15)	
H7	−0.3965	0.2494	0.5570	0.108*	
C8	0.2201 (3)	0.3285 (2)	0.6455 (3)	0.0548 (9)	
C9	0.2891 (3)	0.3544 (2)	0.7448 (3)	0.0543 (9)	
C10	0.3280 (3)	0.4320 (2)	0.7563 (3)	0.0696 (11)	
H10	0.3137	0.4664	0.7023	0.083*	
C11	0.3871 (4)	0.4575 (3)	0.8471 (4)	0.0825 (14)	
H11	0.4141	0.5087	0.8540	0.099*	
C12	0.4065 (4)	0.4069 (3)	0.9284 (4)	0.0781 (13)	
H12	0.4451	0.4244	0.9901	0.094*	
C13	0.3686 (4)	0.3309 (3)	0.9178 (3)	0.0744 (12)	
H13	0.3821	0.2968	0.9721	0.089*	
C14	0.3098 (3)	0.3052 (2)	0.8252 (3)	0.0616 (10)	
H14	0.2843	0.2537	0.8182	0.074*	
C15	−0.0037 (3)	0.1604 (2)	0.3175 (2)	0.0522 (9)	
H15	−0.0264	0.2125	0.3107	0.063*	
C16	−0.0304 (3)	0.1116 (2)	0.2362 (3)	0.0562 (9)	
H16	−0.0706	0.1309	0.1760	0.067*	
C17	0.0021 (3)	0.0342 (2)	0.2435 (2)	0.0545 (9)	
C18	−0.0228 (4)	−0.0190 (3)	0.1528 (3)	0.0783 (13)	
H18A	−0.0952	−0.0030	0.1120	0.117*	
H18B	−0.0310	−0.0717	0.1751	0.117*	
H18C	0.0420	−0.0161	0.1139	0.117*	
C19	0.0616 (3)	0.0085 (2)	0.3355 (2)	0.0499 (8)	
H19	0.0845	−0.0434	0.3437	0.060*	
C20	0.0867 (3)	0.06038 (19)	0.4148 (2)	0.0423 (8)	
C21	0.1553 (3)	0.03960 (19)	0.5139 (2)	0.0431 (8)	
C22	0.1997 (3)	−0.0338 (2)	0.5383 (2)	0.0480 (8)	
H22	0.1856	−0.0741	0.4916	0.058*	
C23	0.2658 (3)	−0.0484 (2)	0.6329 (3)	0.0509 (9)	
C24	0.3132 (4)	−0.1282 (2)	0.6618 (3)	0.0672 (11)	
H24A	0.3985	−0.1262	0.6768	0.101*	
H24B	0.2909	−0.1637	0.6070	0.101*	
H24C	0.2805	−0.1459	0.7202	0.101*	
C25	0.2833 (3)	0.0141 (2)	0.6973 (3)	0.0624 (10)	
H25	0.3281	0.0076	0.7605	0.075*	
C26	0.2360 (3)	0.0853 (2)	0.6704 (3)	0.0589 (10)	
H26	0.2486	0.1260	0.7165	0.071*	
O5	0.0289 (3)	0.29651 (13)	0.46085 (19)	0.0677 (8)	
H5A	0.0560	0.3350	0.4924	0.102*	
O1W	0.3806 (6)	0.4206 (5)	0.4579 (5)	0.116 (3)	0.50
H1WA	0.4507	0.4049	0.4531	0.139*	0.50
H1WB	0.3631	0.3985	0.5107	0.139*	0.50
O2W	0.3699 (7)	0.5847 (4)	0.4468 (5)	0.112 (2)	0.50
H2WA	0.3466	0.5477	0.4814	0.135*	0.50
H2WB	0.3060	0.6076	0.4216	0.135*	0.50

### Atomic displacement parameters (Å^2^)

**Table:**

	*U*^11^	*U*^22^	*U*^33^	*U*^12^	*U*^13^	*U*^23^
Cu1	0.0706 (3)	0.0357 (3)	0.0434 (3)	−0.0046 (2)	0.0005 (2)	−0.00101 (19)
N1	0.0625 (17)	0.0394 (15)	0.0420 (15)	−0.0075 (14)	0.0068 (13)	−0.0005 (13)
N2	0.0549 (16)	0.0372 (15)	0.0427 (15)	−0.0046 (14)	0.0066 (13)	0.0021 (13)
O1	0.0714 (18)	0.0529 (16)	0.0737 (18)	0.0049 (14)	0.0140 (14)	−0.0017 (13)
O2	0.086 (2)	0.089 (2)	0.076 (2)	0.004 (2)	−0.0082 (17)	0.0052 (18)
O3	0.0863 (18)	0.0440 (16)	0.0535 (15)	−0.0054 (14)	−0.0096 (13)	−0.0086 (12)
O4	0.113 (2)	0.0570 (17)	0.0635 (17)	−0.0284 (17)	−0.0080 (16)	0.0101 (15)
C1	0.076 (3)	0.052 (2)	0.053 (2)	−0.003 (2)	−0.011 (2)	−0.018 (2)
C2	0.059 (2)	0.065 (3)	0.070 (3)	0.000 (2)	0.002 (2)	−0.036 (2)
C3	0.073 (3)	0.063 (3)	0.077 (3)	−0.003 (2)	0.010 (2)	−0.013 (2)
C4	0.098 (4)	0.079 (3)	0.090 (3)	−0.009 (3)	0.028 (3)	−0.021 (3)
C5	0.086 (4)	0.121 (5)	0.095 (4)	−0.012 (3)	0.028 (3)	−0.031 (4)
C6	0.062 (3)	0.143 (6)	0.096 (4)	0.006 (3)	0.005 (3)	−0.041 (4)
C7	0.072 (3)	0.118 (4)	0.075 (3)	0.007 (3)	−0.006 (2)	−0.028 (3)
C8	0.062 (2)	0.048 (2)	0.055 (2)	−0.008 (2)	0.0096 (18)	−0.0103 (19)
C9	0.052 (2)	0.051 (2)	0.060 (2)	−0.0014 (18)	0.0075 (17)	−0.0180 (19)
C10	0.071 (3)	0.056 (2)	0.080 (3)	−0.007 (2)	0.005 (2)	−0.017 (2)
C11	0.069 (3)	0.068 (3)	0.106 (4)	−0.011 (2)	−0.001 (3)	−0.041 (3)
C12	0.060 (3)	0.094 (4)	0.077 (3)	0.002 (3)	−0.003 (2)	−0.042 (3)
C13	0.065 (3)	0.092 (4)	0.064 (3)	0.012 (3)	0.000 (2)	−0.012 (3)
C14	0.060 (2)	0.059 (3)	0.065 (2)	0.005 (2)	0.0040 (19)	−0.012 (2)
C15	0.065 (2)	0.043 (2)	0.047 (2)	0.0012 (18)	0.0028 (17)	0.0039 (17)
C16	0.066 (2)	0.056 (2)	0.043 (2)	0.000 (2)	−0.0053 (17)	0.0024 (17)
C17	0.062 (2)	0.053 (2)	0.047 (2)	−0.0061 (19)	0.0019 (16)	−0.0065 (18)
C18	0.106 (3)	0.070 (3)	0.054 (2)	0.006 (3)	−0.006 (2)	−0.017 (2)
C19	0.058 (2)	0.0419 (19)	0.049 (2)	0.0008 (17)	0.0061 (16)	0.0009 (16)
C20	0.0477 (18)	0.0387 (18)	0.0416 (18)	−0.0057 (16)	0.0102 (14)	−0.0001 (15)
C21	0.0487 (18)	0.0388 (18)	0.0427 (18)	−0.0083 (16)	0.0096 (14)	0.0025 (15)
C22	0.057 (2)	0.0390 (19)	0.0483 (19)	−0.0057 (17)	0.0074 (15)	0.0050 (16)
C23	0.055 (2)	0.046 (2)	0.052 (2)	−0.0039 (18)	0.0106 (16)	0.0110 (17)
C24	0.076 (3)	0.057 (2)	0.068 (3)	0.011 (2)	0.009 (2)	0.019 (2)
C25	0.078 (3)	0.060 (3)	0.044 (2)	−0.009 (2)	−0.0075 (18)	0.0094 (19)
C26	0.084 (3)	0.048 (2)	0.042 (2)	−0.009 (2)	−0.0007 (18)	0.0015 (17)
O5	0.107 (2)	0.0367 (14)	0.0542 (15)	−0.0051 (15)	−0.0079 (14)	0.0008 (12)
O1W	0.115 (5)	0.130 (7)	0.119 (6)	0.009 (5)	0.069 (5)	0.051 (5)
O2W	0.152 (6)	0.083 (5)	0.101 (5)	−0.008 (5)	0.013 (5)	0.017 (4)

### Geometric parameters (Å, °)

**Table:**

Cu1—O3	1.955 (2)	C12—C13	1.373 (6)
Cu1—O5	1.997 (2)	C12—H12	0.9300
Cu1—N2	1.999 (3)	C13—C14	1.396 (5)
Cu1—N1	1.999 (3)	C13—H13	0.9300
Cu1—O1	2.219 (3)	C14—H14	0.9300
N1—C21	1.346 (4)	C15—C16	1.374 (5)
N1—C26	1.348 (4)	C15—H15	0.9300
N2—C15	1.338 (4)	C16—C17	1.378 (5)
N2—C20	1.352 (4)	C16—H16	0.9300
O1—C1	1.249 (5)	C17—C19	1.391 (4)
O2—C1	1.258 (5)	C17—C18	1.514 (5)
O2—H2	0.8200	C18—H18A	0.9600
O3—C8	1.276 (5)	C18—H18B	0.9600
O4—C8	1.237 (5)	C18—H18C	0.9600
C1—C2	1.521 (6)	C19—C20	1.385 (4)
C2—C3	1.367 (6)	C19—H19	0.9300
C2—C7	1.396 (6)	C20—C21	1.484 (4)
C3—C4	1.381 (6)	C21—C22	1.378 (5)
C3—H3	0.9300	C22—C23	1.402 (5)
C4—C5	1.361 (7)	C22—H22	0.9300
C4—H4	0.9300	C23—C25	1.373 (5)
C5—C6	1.347 (8)	C23—C24	1.502 (5)
C5—H5	0.9300	C24—H24A	0.9600
C6—C7	1.404 (7)	C24—H24B	0.9600
C6—H6	0.9300	C24—H24C	0.9600
C7—H7	0.9300	C25—C26	1.363 (5)
C8—C9	1.514 (5)	C25—H25	0.9300
C9—C14	1.362 (5)	C26—H26	0.9300
C9—C10	1.404 (5)	O5—H5A	0.8200
C10—C11	1.375 (6)	O1W—H1WA	0.8500
C10—H10	0.9300	O1W—H1WB	0.8500
C11—C12	1.386 (7)	O2W—H2WA	0.8500
C11—H11	0.9300	O2W—H2WB	0.8500
			
O3—Cu1—O5	94.85 (10)	C13—C12—H12	120.0
O3—Cu1—N2	159.99 (11)	C11—C12—H12	120.0
O5—Cu1—N2	91.90 (10)	C12—C13—C14	119.7 (5)
O3—Cu1—N1	90.50 (10)	C12—C13—H13	120.2
O5—Cu1—N1	171.29 (11)	C14—C13—H13	120.2
N2—Cu1—N1	80.85 (11)	C9—C14—C13	121.0 (4)
O3—Cu1—O1	97.39 (11)	C9—C14—H14	119.5
O5—Cu1—O1	90.31 (12)	C13—C14—H14	119.5
N2—Cu1—O1	101.40 (10)	N2—C15—C16	122.6 (3)
N1—Cu1—O1	95.82 (10)	N2—C15—H15	118.7
C21—N1—C26	117.6 (3)	C16—C15—H15	118.7
C21—N1—Cu1	115.4 (2)	C15—C16—C17	120.3 (3)
C26—N1—Cu1	127.0 (2)	C15—C16—H16	119.8
C15—N2—C20	118.1 (3)	C17—C16—H16	119.8
C15—N2—Cu1	126.8 (2)	C16—C17—C19	117.3 (3)
C20—N2—Cu1	115.1 (2)	C16—C17—C18	120.4 (3)
C1—O1—Cu1	128.1 (3)	C19—C17—C18	122.2 (4)
C1—O2—H2	109.5	C17—C18—H18A	109.5
C8—O3—Cu1	128.6 (2)	C17—C18—H18B	109.5
O1—C1—O2	124.4 (4)	H18A—C18—H18B	109.5
O1—C1—C2	117.8 (4)	C17—C18—H18C	109.5
O2—C1—C2	117.7 (4)	H18A—C18—H18C	109.5
C3—C2—C7	118.9 (4)	H18B—C18—H18C	109.5
C3—C2—C1	120.0 (4)	C20—C19—C17	120.0 (3)
C7—C2—C1	121.1 (4)	C20—C19—H19	120.0
C2—C3—C4	121.2 (5)	C17—C19—H19	120.0
C2—C3—H3	119.4	N2—C20—C19	121.7 (3)
C4—C3—H3	119.4	N2—C20—C21	114.3 (3)
C5—C4—C3	119.8 (5)	C19—C20—C21	123.9 (3)
C5—C4—H4	120.1	N1—C21—C22	121.8 (3)
C3—C4—H4	120.1	N1—C21—C20	114.2 (3)
C6—C5—C4	120.7 (5)	C22—C21—C20	124.0 (3)
C6—C5—H5	119.6	C21—C22—C23	120.5 (3)
C4—C5—H5	119.6	C21—C22—H22	119.7
C5—C6—C7	120.6 (5)	C23—C22—H22	119.7
C5—C6—H6	119.7	C25—C23—C22	116.2 (3)
C7—C6—H6	119.7	C25—C23—C24	122.3 (3)
C2—C7—C6	118.9 (5)	C22—C23—C24	121.5 (3)
C2—C7—H7	120.6	C23—C24—H24A	109.5
C6—C7—H7	120.6	C23—C24—H24B	109.5
O4—C8—O3	125.0 (3)	H24A—C24—H24B	109.5
O4—C8—C9	119.7 (3)	C23—C24—H24C	109.5
O3—C8—C9	115.3 (3)	H24A—C24—H24C	109.5
C14—C9—C10	119.0 (4)	H24B—C24—H24C	109.5
C14—C9—C8	121.5 (3)	C26—C25—C23	121.1 (3)
C10—C9—C8	119.4 (4)	C26—C25—H25	119.5
C11—C10—C9	120.3 (4)	C23—C25—H25	119.5
C11—C10—H10	119.9	N1—C26—C25	122.7 (4)
C9—C10—H10	119.9	N1—C26—H26	118.6
C10—C11—C12	120.0 (4)	C25—C26—H26	118.6
C10—C11—H11	120.0	Cu1—O5—H5A	109.5
C12—C11—H11	120.0	H1WA—O1W—H1WB	104.5
C13—C12—C11	120.0 (4)	H2WA—O2W—H2WB	104.5
			
O3—Cu1—N1—C21	−164.4 (2)	O4—C8—C9—C10	−3.7 (6)
N2—Cu1—N1—C21	−2.5 (2)	O3—C8—C9—C10	176.1 (3)
O1—Cu1—N1—C21	98.2 (2)	C14—C9—C10—C11	0.7 (6)
O3—Cu1—N1—C26	15.3 (3)	C8—C9—C10—C11	177.6 (4)
N2—Cu1—N1—C26	177.2 (3)	C9—C10—C11—C12	−1.3 (6)
O1—Cu1—N1—C26	−82.2 (3)	C10—C11—C12—C13	1.2 (7)
O3—Cu1—N2—C15	−111.7 (4)	C11—C12—C13—C14	−0.5 (6)
O5—Cu1—N2—C15	−1.9 (3)	C10—C9—C14—C13	0.0 (6)
N1—Cu1—N2—C15	−177.1 (3)	C8—C9—C14—C13	−176.9 (3)
O1—Cu1—N2—C15	88.8 (3)	C12—C13—C14—C9	0.0 (6)
O3—Cu1—N2—C20	68.5 (4)	C20—N2—C15—C16	0.3 (5)
O5—Cu1—N2—C20	178.3 (2)	Cu1—N2—C15—C16	−179.4 (3)
N1—Cu1—N2—C20	3.2 (2)	N2—C15—C16—C17	−0.2 (6)
O1—Cu1—N2—C20	−91.0 (2)	C15—C16—C17—C19	0.3 (5)
O3—Cu1—O1—C1	100.1 (3)	C15—C16—C17—C18	−177.5 (4)
O5—Cu1—O1—C1	5.2 (3)	C16—C17—C19—C20	−0.5 (5)
N2—Cu1—O1—C1	−86.8 (3)	C18—C17—C19—C20	177.2 (4)
N1—Cu1—O1—C1	−168.6 (3)	C15—N2—C20—C19	−0.6 (5)
O5—Cu1—O3—C8	−13.1 (3)	Cu1—N2—C20—C19	179.2 (2)
N2—Cu1—O3—C8	96.2 (4)	C15—N2—C20—C21	176.9 (3)
N1—Cu1—O3—C8	160.0 (3)	Cu1—N2—C20—C21	−3.3 (3)
O1—Cu1—O3—C8	−104.0 (3)	C17—C19—C20—N2	0.7 (5)
Cu1—O1—C1—O2	−0.6 (5)	C17—C19—C20—C21	−176.6 (3)
Cu1—O1—C1—C2	179.9 (2)	C26—N1—C21—C22	1.6 (5)
O1—C1—C2—C3	26.3 (5)	Cu1—N1—C21—C22	−178.6 (2)
O2—C1—C2—C3	−153.2 (4)	C26—N1—C21—C20	−178.2 (3)
O1—C1—C2—C7	−155.6 (4)	Cu1—N1—C21—C20	1.5 (3)
O2—C1—C2—C7	24.9 (5)	N2—C20—C21—N1	1.2 (4)
C7—C2—C3—C4	0.0 (6)	C19—C20—C21—N1	178.7 (3)
C1—C2—C3—C4	178.2 (4)	N2—C20—C21—C22	−178.7 (3)
C2—C3—C4—C5	−0.4 (7)	C19—C20—C21—C22	−1.2 (5)
C3—C4—C5—C6	1.1 (8)	N1—C21—C22—C23	−1.2 (5)
C4—C5—C6—C7	−1.3 (9)	C20—C21—C22—C23	178.6 (3)
C3—C2—C7—C6	−0.2 (7)	C21—C22—C23—C25	−0.3 (5)
C1—C2—C7—C6	−178.3 (4)	C21—C22—C23—C24	178.9 (3)
C5—C6—C7—C2	0.8 (8)	C22—C23—C25—C26	1.4 (5)
Cu1—O3—C8—O4	−5.3 (6)	C24—C23—C25—C26	−177.8 (3)
Cu1—O3—C8—C9	174.9 (2)	C21—N1—C26—C25	−0.5 (5)
O4—C8—C9—C14	173.1 (4)	Cu1—N1—C26—C25	179.8 (3)
O3—C8—C9—C14	−7.0 (5)	C23—C25—C26—N1	−1.0 (6)

### Hydrogen-bond geometry (Å, °)

**Table:**

*D*—H···*A*	*D*—H	H···*A*	*D*···*A*	*D*—H···*A*
O2W—H2WB···O2^i^	0.85	2.51	3.195 (8)	138
O2W—H2WA···O1W	0.85	2.24	2.821 (10)	125
O1W—H1WB···O4	0.85	2.26	2.932 (6)	136
O1W—H1WA···O2W^ii^	0.85	2.28	2.937 (10)	134
O5—H5A···O4	0.82	1.93	2.642 (4)	145
O2—H2···O5	0.82	1.86	2.636 (5)	158

Symmetry codes: (i) −*x*, −*y*+1, −*z*+1; (ii) −*x*+1, −*y*+1, −*z*+1.
